# *n*-3 PUFA added to high-fat diets affect differently adiposity and inflammation when carried by phospholipids or triacylglycerols in mice

**DOI:** 10.1186/1743-7075-10-23

**Published:** 2013-02-15

**Authors:** Manar Awada, Anne Meynier, Christophe O Soulage, Lilas Hadji, Alain Géloën, Michèle Viau, Lucie Ribourg, Berengère Benoit, Cyrille Debard, Michel Guichardant, Michel Lagarde, Claude Genot, Marie-Caroline Michalski

**Affiliations:** 1 INRA, U1362, CarMeN, Villeurbanne, F-69621, France; 2 INSA-Lyon, IMBL, Villeurbanne, F-69621, France; 3 INRA, UR1268 BIA, Nantes, F-44316, France; 4 INSERM U1060, CarMeN, Oullins, F-69921, France; 5 Université de Lyon, Villeurbanne, F-69622, France; 6 INRA USC1362, INSERM U1060, Cardiovasculaire Métabolisme diabétologie et Nutrition, CarMeN Laboratory, InFoLip team, IMBL Building, INSA-Lyon, 11 avenue Jean Capelle, Villeurbanne cedex, 69621, France

**Keywords:** *n*-3 PUFA, Phospholipid, Triacylglycerol, High-fat diet, Inflammation, Oxidative stress, Adipose tissue

## Abstract

**Background:**

Dietary intake of *n*-3 polyunsaturated fatty acids (PUFA) is primarily recognized to protect against cardiovascular diseases, cognitive dysfunctions and the onset of obesity and associated metabolic disorders. However, some of their properties such as bioavailability can depend on their chemical carriers. The objective of our study was to test the hypothesis that the nature of *n*-3 PUFA carrier results in different metabolic effects related to adiposity, oxidative stress and inflammation.

**Methods:**

4 groups of C57BL/6 mice were fed for 8 weeks low fat (LF) diet or high-fat (HF, 20%) diets. Two groups of high-fat diets were supplemented with long-chain *n*-3 PUFA either incorporated in the form of phospholipids (HF-ω3PL) or triacylglycerols (HF-ω3TG).

**Results:**

Both HF-ω3PL and HF-ω3TG diets reduced the plasma concentrations of (i) inflammatory markers such as monocyte chemoattractant protein-1 (MCP-1) and interleukin 6 (IL-6), (ii) leptin and (iii) 4-hydroxy-2-nonenal (4-HNE), a marker of *n*-6 PUFA-derived oxidative stress compared with the control HF diet. Moreover, in both HF-ω3PL and HF-ω3TG groups, MCP-1 and IL-6 gene expressions were decreased in epididymal adipose tissue and the mRNA level of gastrointestinal glutathione peroxidase GPx2, an antioxidant enzyme, was decreased in the jejunum compared with the control HF diet. The type of *n*-3 PUFA carrier affected other outcomes. The phospholipid form of *n*-3 PUFA increased the level of tocopherols in epididymal adipose tissue compared with HF-ω3TG and resulted in smaller adipocytes than the two others HF groups. Adipocytes in the HF-ω3PL and LF groups were similar in size distribution.

**Conclusion:**

Supplementation of mice diet with long-chain *n*-3 PUFA during long-term consumption of high-fat diets had the same lowering effects on inflammation regardless of triacyglycerol or phospholipid carrier, whereas the location of these fatty acids on a PL carrier had a major effect on decreasing the size of adipocytes that was not observed with the triacyglycerol carrier. Altogether, these results would support the development functional foods containing LC *n*-3 PUFA in the form of PL in order to prevent some deleterious outcomes associated with the development of obesity.

## Background

Chronic inflammation and oxidative stress are recognized as major factors involved in the pathogenesis of several current metabolic diseases such as obesity, type 2 diabetes and cardiovascular diseases (CVD) [1-3]. Elevated levels of pro-inflammatory cytokines and chemokines, such as interleukins (IL) and monocyte chemotactic protein-1 (MCP-1), are hallmarks of the metabolic syndrome [1,2]. It is now established that white adipose tissue (WAT) is not only an energy-storage tissue but also an endocrine organ that secretes various bioactive molecules namely “adipokines” including adiponectin, leptin, IL-6 and MCP-1, [4]. These molecules have a key role in the regulation of systemic and energy metabolism. Indeed, dysregulated production of these adipokines due to WAT dysfunction and excess adiposity can contribute to the pathogenesis of obesity and insulin resistance [5]. High-fat intake has been repeatedly shown to play a significant part in the obesity-associated low grade chronic inflammation, which is characterized in human and mice by macrophage infiltration in WAT [6-9].

In this context, dietary supplementation with long-chain *n*-3 polyunsaturated fatty acids (LC *n*-3 PUFA), in particular docosahexaenoic acid (DHA; 22:6 *n*-3) and eicosapentaenoic acid (EPA; 20:5 *n*-3) presents a variety of health benefits. These LC *n*-3 PUFA are known to be protective by reducing inflammation in both blood and WAT [6,10,11], improving lipid metabolism [12], decreasing risk of CVD [13,14] and several neurodegerenative diseases [15,16]. Therefore, nutritional recommendations of 250 mg/day of EPA/DHA have been established in Western societies for *n*-3 PUFA intake, to achieve nutrient adequacy and lower *n*-6/*n*-3 PUFA ratio [17].

Dietary EPA and DHA are provided mostly by fatty fish, where they are mainly esterified in triacylglycerols (TG). In other sources such as krill, the major proportion of these LC *n*-3 PUFA is esterified in phospholipids (PL). Of note, compared with fish oil, krill oil contains the same amount of DHA but a greater proportion of EPA [18]. The latter is a major precursor of anti-inflammatory eicosanoids [19]. Some authors have compared the potential effects of different LC *n*-3 PUFA formulations on lipid metabolism. The supplementation of LC *n*-3 PUFA in the form of PL would exert superior biological and nutritional functions compared to TG. This would include (i) anti-inflammatory actions [20,21] and antioxidant activities on the brain lipids [22], (ii) improved memory and learning [23], (iii) reduced blood and tissue lipids [24-26], (iv) increased bioavailability of EPA and DHA in plasma [27,28] and (v) tendency to reduce obesity [21]. In humans, the effects of PL-bound LC *n*-3 PUFA provided by krill oil on plasma lipids were similar to those of TG-bound LC *n*-3 PUFA from fish oil but at lower dose of LC *n*-3 PUFA [27]. In the rodent studies, the amount of LC *n*-3 PUFA in the diets were usually high, i.e. ~ 8–15% of total fats [21,26,29].

To date, available data are insufficient to assess whether the supplementation of Western-type high-fat (HF) diets with LC *n*-3 PUFA carried either by TG or by PL can make any difference in the metabolic outcomes, with an aim to prevent or treat metabolic disorders.

The aim of the present study was to test the hypothesis that long-term intake of nutritional amounts of *n*-3 PUFA in realistic HF diets could exert different effects according to the PL or TG carrier on (i) the development of adiposity and (ii) associated oxidative stress and inflammation.

## Methods

### Materials

Omegavie® Tuna oil 25 DHA flavorless was provided by Polaris (Pleuven, France) as a source of triacylglycerol rich in LC *n*-3 fatty acids. A lecithin rich in DHA was enzymatically synthesized by Polaris (Pleuven, France) and further purified by precipitation with cold acetone to eliminate all traces of triacylglycerols and ethyl esters [30]. As shown by HPLC paired with evaporative light-scattering detector and external calibration curves, PL-DHA was composed of lysophosphatidylcholine (lysoPC: 49.2 ± 0.7 wt%), phosphatidylcholine (PC; 33.5 ± 0.4 wt%), phosphatidylinositol (PI; 9.0 ± 0.3 wt%), phosphatidylethanolamine (PE: 4.8 ± 0.7 wt%) and sphingomyelin (3.5 ± 0.2 wt%). Lard was supplied by Celys (Rezé, France) and kiwi seed oil, as additional source of alpha-linolenic acid, by Polaris (Pleuven, France). Sunflower oil (Lesieur®) was purchased from a local supermarket and oleic sunflower oil from Olvéa (Marseille, France). The vegetable lecithin rich in linoleic acid 18:2 *n*-6 (PL-LA) was sold by Lipoid (Ludwigshafen, Germany) and was composed exclusively of phosphatidylcholine and antioxidants (no TAG).

### Preparation of lipid for mice diets

Low fat (LF), HF and the two HF lipid blends containing LC *n*-3 PUFA in the form of PL or TG were prepared at the labscale. DHA and, in lower amount, EPA were supplied either in the form of triacylglycerols (HF-ω3TG) or phospholipids (HF-ωPL). The fatty acid compositions of both the tuna oil and the lecithin rich DHA product are reported in Additional file 1. The four blends were prepared by direct mixing of the different oils and fat sources, including the lard in the proportions indicated in Table 1. Then they were stored under a flux of nitrogen at −20°C and sent to SAFE (Augy, France) for preparing the mice diets. The composition of the four diets is reported Table 1. HF-ω3TG and HF-ω3PL are the diets that served as unoxidized *n*-3 diet controls in our other study about the impact of PUFA oxidation [30].

**Table 1:** Formulation of the experimental diets

**High-fat containing n-3 PUFA as**	**LF**	**HF**	**HF-ω3PL**	**HF-ω3TG**
**Ingredient (g/100 g)**				
Lipid mixture	5	20	20	20
Among which:				
Lard	2	18.10	18.10	18.06
Sunflower oil	0.64	1.1	0.6	0.2
Oleic sunflower	1.27	0.0	0.4	-
Kiwi seed oil	0.29	0.0	0.1	0.02
Tuna oil	-	-	-	0.9
Phospholipids				
PL-DHA	-	-	0.8	-
Lecithin PL-LA	0.8	0.8	-	0.8
Corn starch	54	39	39	39
Casein	20	20	20	20
Sucrose	10	10	10	10
Pure cellulose	5	5	5	5
Vitamin mixture	5	5	5	5
Mineral mixture	1	1	1	1
Tocopherols^a^	0.000	0.084	0.099	0.092
Energy content (kJ/g)	14.88	18.14	18.14	18.14
Energy%				
Protein	19.1	15.7	15.7	15.7
Carbohydrates	57.6	34.1	34.1	34.1
Lipids	12.8	41.5	41.5	41.5

^a^Considering the difference observed in tocopherols in lipid mixtures, which can affect their metabolic impact [31], care was taken to supplement lipid mixtures with α-tocopherol during formulation to achieve iso-tocopherol diets.

### Animals and diets

Male C57BL/6 mice (8 wk, 20 g) from Janvier SA (Le Genest Saint-Isle, France) were housed in a temperature-controlled room (22°C) with a 12 h light/12 h dark cycles. After 2 weeks of chow diet, mice were randomly divided into four groups fed one of the four following diets for 8 weeks: LF, HF, HF-ω3TG and HF-ω3PL diets. Animal experiments were performed under the authorization n°69-266-0501 (Direction Départementale des Services Vétérinaires du Rhône, France). All experiments were carried out in compliance with the French Ministry of Agriculture guidelines (n° 87–848) and the E.U. Council Directive for the Care and Use of Laboratory Animals of November 24th, 1986 (86/609/EEC), in conformity with the Public Health Service (PHS) Policy on Humane Care and Use of Laboratory Animals. COS holds a special licence (n° 69266257) to experiment on living vertebrates issued by the French Ministry of Agriculture and Veterinary Service Department. Body weight was measured twice a week and food intake was measured weekly. After 8 weeks, mice were euthanized by intraperitoneal (IP) injection of sodium pentobarbital and blood was collected by cardiac puncture under pyrogen-free conditions on heparin-containing tubes. Plasma, liver, WAT, muscles, duodenum and jejunum were collected. For analysis of 4-hydroxy-2-alkenals, due to plasma volume constraints, 3 pools of 300 μL obtained from 3 mice (100 μL per mice) were used for each group.

### 4-Hydroxy-2-alkenals: derivatization, analysis and quantification

4-hydroxy-2-alkenals were derivatized from 300 μl of plasma as described previously [32]. Deuterated 4-hydroxy-2-nonenal (4-HNE) and 4-hydroxy-2-hexenal (4-HHE) (20 ng) used as internal standards were added to the samples. Briefly, they were treated with O-2,3,4,5,6-pentafluorobenzyl hydroxylamine hydrochloride. After acidification with H_2_SO_4_, pentafluorobenzyloxime derivatives were extracted with methanol and hexane. The hydroxyl group was then converted into trimethylsilylether after an overnight treatment with N,O-bis(trimethylsilyl)trifluoroacetamide at room temperature. The pentafluorobenzyloxime trimethylsilylether derivatives of 4-HHE (O-PFB-TMS-4-HHE) and 4-HNE (O-PFB-TMS-4-HNE) were then analyzed by GC-MS using negative ion chemical ionization (NICI) mode on a Hewlett-Packard quadripole mass spectrometer interfaced [32] with a Hewlett-Packard gas chromatograph (Les Ullis, France). For the determination of 4-hydroxy-2-alkenals in liver, the tissue was firstly minced in liquid nitrogen with PIPES, then centrifuged and the supernatant treated as previously described. Regarding WAT, a protocol has been adapted to the extraction and derivatization of 4-hydroxy-2-alkenals in lipophilic media. Briefly the o-pentafluorobenzyl hydroxylamine was prepared by dilution in hexane containing pure triethylamine leading to the formation of a precipitate, which was removed. Oxime derivatives were then prepared and extracted with hexane. Lipids were remove by solid phase extraction on silica cartridge, and the oxime derivatives were eluated with hexane/diethyl ether (50/50, vol/vol). The second derivatization (silylation) was achieved as previously described. The method allowed the quantification of free 4-HHE and 4-HNE.

### sCD14, LBP, IL-6, MCP-1, leptin, adiponectin, insulin and glucose measurements

MCP-1, IL-6 (Clinisciences, France), sCD14, LBP (Enzo Life Sciences), leptin, adiponectin (SpiBio, Montigny Le Bretonneux, France) and insulin (Crystal Chem Inc., USA) plasma levels were assayed by ELISA kits according to the manufacturer’s instructions. Blood glucose levels were measured on animals in mouse-tail blood using a glucometer (Accu-Chek ®, Roche, France).

### Plasma triacylglycerols and NEFA measurements

Plasma triacyglycerols (TAG) were measured with the triglyceride PAP kit (BioMérieux France) as previously described [33]. Plasma TAG concentration was calculated by subtracting the free glycerol in plasma measured with the glycerol UV-method (R-Biopharm/Boehringer, Mannheim, Germany). Plasma NEFA was measured using NEFA-C kit (Wako Chemicals, Neuss, Germany).

### Fatty acid analysis

Total lipids were extracted from 35 μL of plasma as described previously [33] and from tissues according to Folch et al. [34]. The organic phase (solvent) was evaporated under N_2_ and total fatty acids were transesterified using boron trifluoride in methanol (BF_3_/methanol) and in the presence of heptadecanoic acid (C17:0, Sigma, France) as an internal standard [33]. The FA methyl esters were then analyzed by GC using a DELSI instrument model DI 200 equipped with a fused silica capillary SP-2380 column (60 m × 0.22 mm).

### Quantification of tocopherols

Tocopherols in epipidymal WAT (eWAT) and retroperitoneal WAT (rWAT) were quantified without saponification according to a method described by [35]. An aliquot of lipid extract was dried under N_2,_ then solubilized in n-hexane and analyzed by HPLC paired with a fluorimeter detector (λex = 295 nm and λem = 330 nm). Separation of the different isomers of tocopherols and tocotrienols was achieved on a polar column (Acclaim Polar advantage II, Dionex, 3 μm; 250 × 3 mm) in isocratic mode at 0.5 ml/min with hexane/methyl-terbutyl-ether 90/10, vol/vol. The quantification was realized by comparison of the peak areas with calibration curves performed with external standard solutions of α, γ, β and δ tocopherols (Calbiochem, tocopherol set) and γ tocotrienol (Sigma, France). An aliquot of each diet was saponified for 30 min at 70°C in a mixture containing potassium hydroxide (50% w/vol in water), pyrogallol in ethanol (1% w/vol). Tocopherols were extracted with hexane containing 0.005% (w/vol) of BHT. After centrifugation and washing, the organic phase was mixed. The solvent was removed under vacuum, and then under a stream of nitrogen. Tocopherols were finally dissolved in *n*-hexane and then quantified as described previously.

### Cellularity study: measurement of adipocyte size and number

Preparation of adipose tissue for determination of cell size was performed essentially as described previously [36]. Briefly, 30–40 mg of eWAT or rWAT was immediately fixed in osmium tetroxide for 96 hours at room temperature. After washing, adipose cell-size distribution was then assessed using a Beckman Coulter Multisizer IV (Beckman Coulter) with a 400 μm aperture. The range of cell-sizes that can effectively be measured using this aperture is 20–240 μm. The instrument was set to count 1000 particles per run, and the fixed-cell suspension was diluted so that coincident counting was less than 10%. After collection of pulse sizes, the data were expressed as particle diameters and displayed as histograms of counts against diameter using linear bins and a linear scale for the cell diameter. Cell-size distributions were drawn from measurement of at least 12 000 cell diameters per animal. Mean cell weight was calculated as measured Cell volume × TG density (0.9); cell number were calculated as Cell weight/WAT mass (eWAT or rWAT).

### Quantitative PCR analysis

Total RNA was extracted from 50 mg of duodenum and jejunum of mice using the NucleoSpin® RNA/Protein kit (Macherey Nagel, Duren, Germany), and from 50 mg of eWAT or rWAT with TRIzol (Invitrogen, Eragny, France). cDNAs were synthesized from 1 μg of total RNA in the presence of 100 units of Superscript II (Invitrogen, France) with a mixture of random hexamers and oligo (dt) primers (Promega, Charbonnières, France). The amount of target mRNAs was measured by RT, followed by real-time PCR, using a Rotor-Gene Q (Qiagen, France). The amount of target mRNAs was measured by RT, followed by real-time PCR, using a Rotor-Gene Q (Qiagen, France). Primer sequence and RT-quantitative PCR conditions are available upon request (cyrille.debard@univ-lyon1.fr). Hypoxanthine guanine phosphoribosyl transferase (HPRT) mRNA was used to normalize data of duodenum, jejunum and WAT of mice.

### Statistical analysis

All data are presented as means ± SEM and were analysed using Statview 5.0 software (Abacus Concept, Berkeley). One-way ANOVA followed by Fisher PLSD was used to compare the four groups. Differences were considered significant at *P* < 0.05.

## Results

### Diet compositions

Table 2 shows that we succeeded in producing diets equilibrated in terms of *n*-3 PUFA supplies and *n*-6/*n*-3 ratios, with LC *n*-*3* PUFA in the form of PL or TG. Importantly, HF-ω3TG diet, HF diet and LF diet also contained PL in the form of PL-LA lecithin, so that effects of PL-ω3 can be attributed to the molecular form of LC *n*-3 PUFA in the diet, not to the presence of phospholipids.

**Table 2:** Fatty acid composition in the diets

**FA mg/g diet**	**LF**	**HF**	**HF-ω3PL**	**HF-ω3TG**
SFA	11.3 ± 1.6	71 ± 12^$^	64 ± 1^$^	66 ± 5^$^
MUFA	24 ± 6	82 ± 14^$^	76 ± 1^$^	72 ± 5^$^
*n*-6 PUFA	12 ± 2	29 ± 5^$^	21 ± 1^$^	21 ± 1^$^
*n*-3 PUFA	2.1 ± 0.2	2.0 ± 0.4	2.8 ± 0.2^$*^	3.9 ± 0.1^$*^
Among which 18 :3 *n*-3	2.1 ± 0.2	2.0 ± 0.4	2.0 ± 0.1	1.9 ± 0.1
20 :5 *n*-3	-	-	0.2 ± 0.0^$*£^	0.4 ± 0.2^$*^
22 :6 *n*-3	-	-	2.8 ± 0.2^$*£^	3.9 ± 0.2^$*^
Total PUFA	13.8 ± 2.3	30.4 ± 5^$^	23.7 ± 0.4^$^	25.0 ± 1.4^$^
*n*-6/*n*-3 ratio	5.4 ± 0.4^*^	14.3 ± 0.3	7.7 ± 0.5^*^	5.4 ± 0.2^*^
Total FA	49 ± 9	184 ± 31^$^	163 ± 2^$^	162 ±11^$^
α-tocopherol (μg/g chow)	185 ± 6	219 ± 4	201 ± 1	207 ± 18

(*P < 0.05 *vs* HF); (^$^P < 0.05 *vs* LF), (^£^P < 0.05 *vs* HF-w3TG). ANOVA followed by Fisher test. Data are mean ± SEM for n = 3. Abbreviations: FA, fatty acids; MUFA, monounsaturated fatty acids; SFA, saturated fatty acids.

The HF-ω3PL diet contained slightly less *n*-3 PUFA than the HF-ω3TG diet, as frequently found in dietary *n*-3 PUFA sources. In *n*-3 PUFA enriched diets, *n*-3 PUFA contents and *n*-6/*n*-3 ratios were consistent with dietary recommendations, whereas *n*-6/*n*-3 ratio was nearly 15 in HF diet as observed in human Western diets.

### PL-ω3 and TG-ω3 in high-fat diet modify biometric parameters

As shown in Table 3, the supplementation of HF diets with LC *n*-3 PUFA decreased the body weight gain in HF-ω3PL fed mice compared to HF mice but not in HF-ω3TG mice. Liver weight was significantly lower in mice fed HF-ω3PL or HF-ω3TG diets compared to HF group, and even lower than LF group regarding HF-ω3PL. White adipose tissue weight (total of retroperitoneal, epididymal and subcutaneous adipose tissues) was also lower in HF-ω3PL group than in HF group but no significant difference was observed between HF-ω3PL and HF-ω3TG groups. However, the lean mass estimated by the weight of *gastrocnemius* muscle was not different between the groups (Table 3).

**Table 3:** Morphologic parameters, food intake and plasma lipid concentrations of mice

**Morphologic parameters**	**Mice groups according to dietary lipids**			
	**LF**	**HF**	**HF-ω3PL**	**HF-ω3TG**
Biometric data				
Initial Body weight (g)	24.1 ± 0.4	24.2 ± 0.3	23.9 ± 0.3	24.5 ± 0.3
Body weight gain (g)	4.1 ± 0.1	4.2 ± 0.5	3.1 ± 0.1^*$^	3.7 ± 0.3
Energy Intake (kJ/mouse/d)	39.1 ± 0.9	68.9 ± 0.3^$^	60.2 ± 1.3^$^	59.8 ± 1.3^$^
Liver weight (g)	1.39 ± 0.03	1.36 ± 0.03	1.21 ± 0.04^*$^	1.27 ± 0.04^*^
WAT weight (g)	0.84 ± 0.05	1.03 ± 0.12	0.72 ± 0.05^*^	0.93 ± 0.11
Gastrocnemius (g)	0.16 ± 0.0	0.16 ± 0.0	0.16 ± 0.0	0.15 ± 0.0
Plasma lipids				
TAG (mM)	0.89 ± 0.06	0.66 ± 0.09^$^	0.64 ± 0.04^$^	0.57 ± 0.04^$^
NEFA (mM)	0.62 ± 0.5	0.35 ± 0.02^$^	0.27 ± 0.01^$^	0.37 ± 0.02^$^
Glucose (mmol/L)	9.1 ± 0.8	8.6 ± 0.5	9.4 ± 0.9	9.2 ± 0.7
Plasma insulin (pmol/L)	37.0 ± 6.9	36.8 ± 3.0	24.5 ± 3.0	26.5 ± 4.8
Liver lipids (mg total fatty acids/g tissue)	30.3 ± 2.9	34.0 ± 3.0	30.3 ± 0.6	33.1 ± 3.7
Lipid peroxidation markers in plasma (nM)				
4-HHE	23.3 ± 13.5	103.7 ± 36.5	88.8 ± 32.0	128.0 ± 37.5
4-HNE	4.9 ± 0.3^*^	13.4 ± 0.5	5.9 ± 1.1^*^	8.6 ± 0.3^*^

(*P < 0.05 *vs* HF); (^$^P < 0.05 *vs* LF), ANOVA followed by Fisher test.

Data are mean ± SEM for n = 8 per group. Abbreviations: WAT, white adipose tissue; TAG, plasma triacyglycerols; NEFA, non-esterified fatty acids; 4-HHE, 4-hydroxy-2-hexenal; 4-HNE, 4-hydroxy-2-nonenal.

For 4-hydroxy-2-alkenals analysis, 3 pools of 300 μL obtained from 3 mice (100 μL per mice) were used for each group (n = 3).

Energy intake was evaluated during the 8 weeks of feeding trial. Table 3 shows that it was higher in the three HF groups than in LF group. However, results did not show any difference between HF-ω3PL and HF-ω3TG groups. After 8 weeks feeding, free fatty acid and triacyglycerol concentrations were lower (*P* < 0.05) in the plasma of the three groups of mice fed HF diets, regardless of supplementation with *n*-3 PUFA, than in LF group (Table 3). Plasma insulin and glucose levels did not differ among the groups. Neither plasma free fatty acid nor triacyglycerol concentrations were significantly different between the three groups of mice fed HF diets. Hepatic lipid accumulation was similar among groups.

### Fatty acid composition in plasma and tissues reflects the enrichment with PL-ω3 and TG-ω3 in the high-fat diets

Fatty acid composition of total plasma, liver and WAT were characterized to investigate whether long-term consumption of different diets could change PUFA metabolism. In fact, the profile of total FA in plasma (Table 4), in liver and eWAT (See Additional file 2) reflected the composition of ingested dietary fats (Table 2). FA profile of rWAT was similar to that of eWAT (See Additional file 3).

**Table 4:** Fatty acid profile in plasma in mice fed different diets

**Major FA in plasma**	**LF**	**HF**	**HF-ω3PL**	**HF-ω3TG**
**Total FA (mol/100 mol FA):**				
SFA	33.7 ± 0.3	34.5 ± 0.2	34.4 ± 1.3	36.1 ± 2.6
16:1 *n*-7	4.0 ± 0.5	1.6 ± 0.2^$^	1.2 ± 0.02^$^	1.6 ± 0.4^$^
18:1 *n*-7	2.7 ± 0.2	1.5 ± 0.1^$^	1.3 ± 0.1^$^	1.4 ± 0.3^$^
18:1 *n*-9	19.3 ± 1.9	15.6 ± 0.7^$^	16.1 ± 1.9^$^	13.4 ± 6.7^$^
MUFA	27.9 ± 1.0	19.6 ± 0.3^$^	19.5 ± 0.2^$^	17.3 ± 6.7^$^
18:2 *n*-6	21.2 ± 0.6	25.4 ± 0.2^$^	26.1 ± 0.4^$^	26.4 ± 0.8^$^
20:4 *n*-6	9.6 ± 1.2^*^	14.3 ± 0.4	11.2 ± 0.5^*^	9.5 ± 0.8^*^
*n*-6	33.1 ± 0.9^*^	42.1 ± 0.5	39.2 ± 1.0^$^	38.2 ± 3.2^$*^
18:3 *n*-3	0.4 ± 0.1	Tr^$^	0.3 ± 0.0^*$^	0.2 ± 0.0^$*^
20:5 *n*-3	0.7 ± 0.0	Tr^$^	0.7 ± 0.0^*£^	1.2 ± 0.1^$*^
22:6 *n*-3	3.8 ± 0.4	3.2 ± 0.1	4.9 ± 0.3^$*£^	6.6 ± 0.5^$*^
*n*-3	5.2 ± 0.4	3.9 ± 0.1^$^	6.4 ± 0.2^*£^	8.4 ± 1.3^$*^
*n*-6/*n*-3 ratio	6.4 ± 0.3^*^	10.9 ± 0.4	6.1 ± 0.2^*^	4.6 ± 0.4^*^

(*P < 0.05 *vs* HF), (^$^P < 0.05 *vs* LF), (^£^P < 0.05 *vs* HF-ω3TG). Data are mean ± SEM for n = 5 per group. Abbreviations: Tr, traces FA; fatty acids; MUFA, monounsaturated fatty acids; SFA, saturated fatty acids.

Table 4 shows that the plasma arachidonic acid (20:4 *n*-6), an *n*-6 FA precursor of pro-inflammatory mediators in biological membranes, was significantly higher in HF group than in three other groups. EPA proportion was higher in plasma of mice fed the HF diets supplemented with *n*-3 PUFA compared with HF group, and higher in HF-ω3TG group than in HF-ω3PL group. Importantly, plasma DHA was higher in the two HF groups supplemented with LC *n*-3 PUFA in the form of PL or TG compared with LF and HF groups. Additionally, the proportions of LC *n*-3 PUFA (EPA and DHA) were higher in the HF-ω3TG group than in the HF-ω3PL group as observed in the diets.

**Figure 1:**
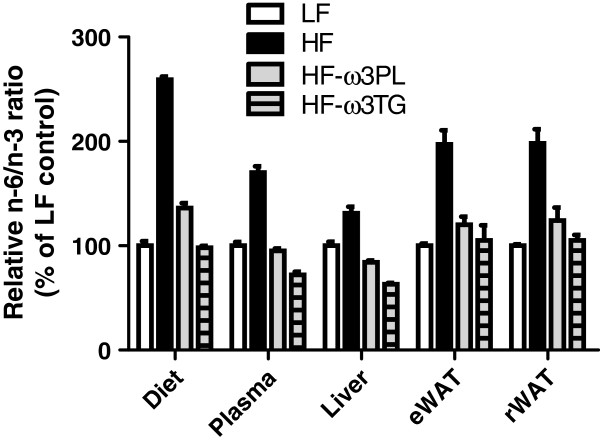
**Relative n-6/n-3 fatty acid ratio in diets, plasma, Liver and WAT.** The mice were fed LF, HF, HF-ω3PL and HF-ω3TG diets. The values were expressed as percentage of the ratio measured in the groups. Data are means ± SEM (n = 3-6).

In liver like in eWAT, DHA and EPA levels were significantly increased in both HF-ω3PL and HF-ω3TG groups compared to LF and HF groups. A significant difference of LC *n*-3 PUFA levels was observed between HF-ω3PL and HF-ω3TG groups (See Additional file 2), as observed in the plasma. The *n*-6/*n*-3 ratio, related to a risk of inflammation derived from PUFA metabolites, was higher in mice fed HF diet than the three other groups. This ratio was not different between HF-ω3PL and HF-ω3TG groups (Table 4 and Additional file 2). The relative difference of *n*-6/*n*-3 ratio that existed between the high-fat diets and the LF diet was altogether still observed in plasma and tissues after 8 weeks of diet (Figure 1). Altogether, both diets enriched in LC *n*-3 PUFA resulted in proper accretion of these FA in tissues.

### Effects of PL-ω3 and TG-ω3 in high-fat diet on plasma markers of inflammation, metabolic endotoxemia and oxidative stress

**Figure 2:**
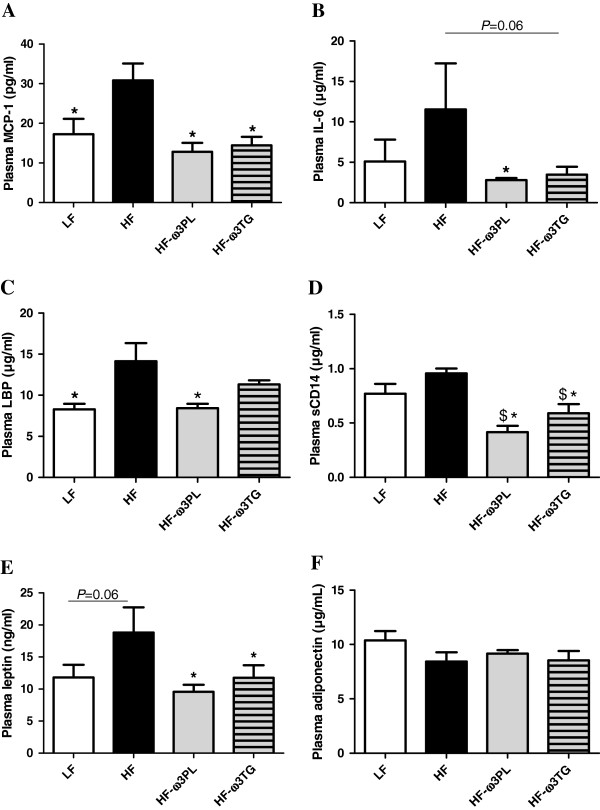
**Inflammation and endotoxin metabolism parameters in plasma of mice fed different diets. **(**A)** monocyte chemoattractant protein-1 (MCP-1; pg/ml); (**B**) Interleukin-6 (IL-6; μg/ml). (**C**) lipopolysaccharide binding protein (LBP; μg/ml). (**D**) (soluble cluster of differentiation 14 (sCD14; μg/ml). (**E**) leptin (ng/ml); (**F**) Adiponectin (μg/ml). Data are means ± SEM (n = 6-8). (*P < 0.05 *vs* HF); (^$^P < 0.05 *vs* LF). ANOVA followed by Fisher test.

High-fat diets are known to induce a metabolic inflammation related to (i) the concentrations of different types of adipokines and (ii) endotoxin transport. Regarding inflammation, we show higher concentrations in plasma of the chemokine MCP-1 (Figure 2A) in HF group than in the three other groups. Plasma interleukin IL-6 was also significantly higher in HF group than in HF-ω3PL group (Figure 2B) and tended to be higher in HF group than in HF-ω3TG mice (*P* = 0.06). Both *n*-3 diets even resulted in plasma concentrations of inflammatory markers similar to the LF diet. Among plasma markers involved in the transport of the pro-inflammatory endotoxins, the lipopolysaccharide binding protein (LBP) level was higher in plasma of HF mice than in LF and HF-ω3PL groups (Figure 2C). However, no difference was found between HF and HF-ω3TG groups or between HF-ω3PL and HF-ω3TG groups. The plasma level of soluble cluster of differentiation 14 (sCD14), an endotoxin receptor, was also higher in HF mice than in the two HF groups supplemented with LC *n*-3 PUFA (Figure 2D). Regarding adipokines in plasma, results showed an increased leptin concentration in the HF group *vs* all other groups (Figure 2E). Supplementation with LC *n*-3 PUFA in both forms allowed recovering leptin concentrations similar to LF group. In contrast, there was no difference in plasma adiponectin concentration among the groups (Figure 2F).

LC PUFA are known to be prone to oxidation because of their high degree of unsaturation. They form oxidized end-products which contribute to oxidative stress. We measured the levels of 4-hydroxy-2-hexenal (4-HHE) and 4-hydroxy-2-nonenal (4-HNE), two markers of lipid peroxidation derived from the oxidation of *n*-3 PUFA and *n*-6 PUFA respectively. As shown in Table 3, the plasma level of 4-HHE did not differ among the groups, although there was large variation within groups. The plasma level of 4-HNE was significantly higher in HF group than in the three other groups albeit remaining low (Table 3). No differences in 4-HHE and 4-HNE concentrations were observed in the liver that is a major target organ for these alkenals (0.5 to 2 nmol/g regardless of group).

### PL-ω3 and TG-ω3 in high-fat diet improve markers related to inflammation and oxidative stress in WAT

**Figure 3:**
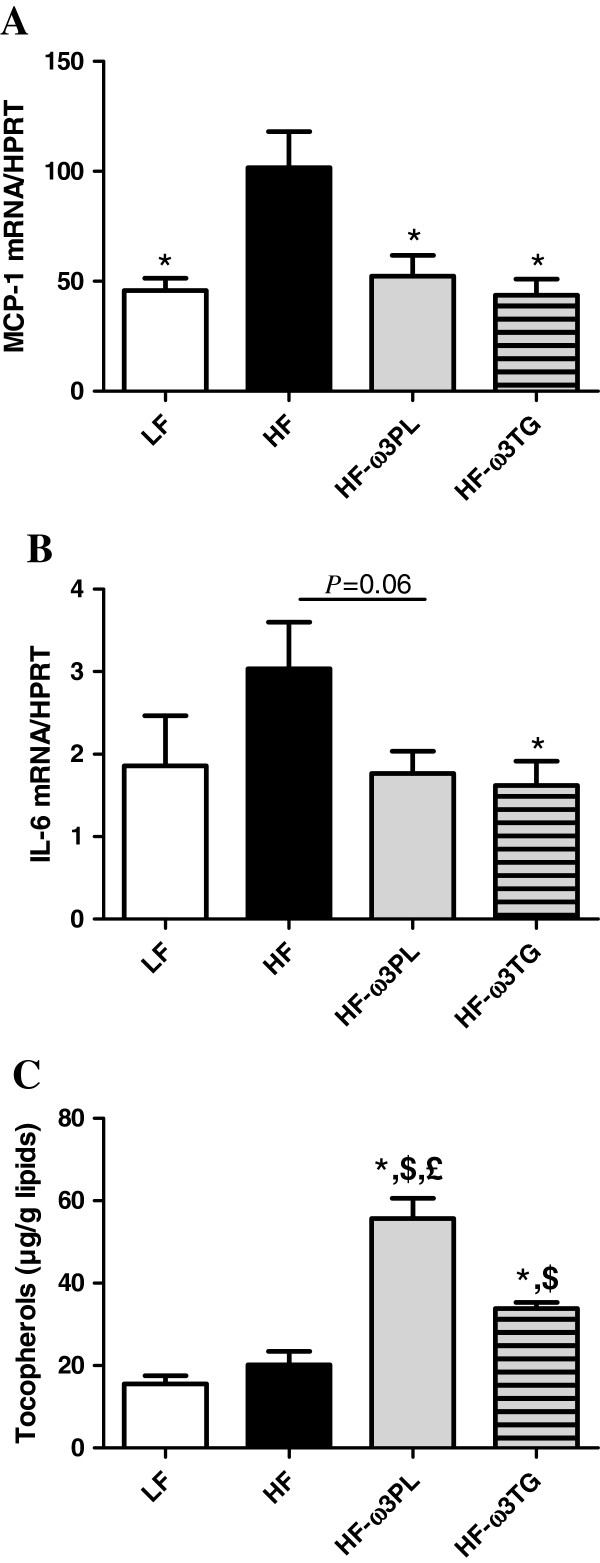
**Inflammatory markers and tocopherol level in epididymal white adipose tissue (eWAT).** MCP1 mRNA (**A**); IL-6 mRNA (**B**) and Tocopherol (μg/g lipids) (**C**). RT-quantitative PCR results of mRNA expression. Tocopherol level was measured as described in Materials & Methods. Bars represents means ± SEM of n = 5-6 mice. (*P < 0.05 *vs* HF); (^$^P < 0.05 *vs* LF); (^£^P < 0.05 *vs* HF-ω3TG). ANOVA followed by Fisher test.

Because we noticed a significant decrease of inflammatory markers in the plasma of mice fed HF diet supplemented with LC *n*-3 PUFA, we examined the MCP-1 and IL-6 gene expression in WAT. MCP-1 expression in eWAT was significantly higher in HF group than in the other groups (Figure 3A, *P* < 0.01). The same difference between HF and HF-ω3PL groups was observed in rWAT (see Additional file 4). The expression of mRNA IL-6 was significantly decreased in HF-ω3TG group compared to HF group and tended to be decreased in HF-ω3PL group compared to HF group (Figure 3B). In addition, the level of tocopherols was higher in eWAT of mice fed HF-ω3PL and HF-ω3TG diets than LF and HF groups (Figure 3C). Noticeably, the level of tocopherols was higher in HF-ω3PL group than in HF-ω3TG group (P < 0.05) despite the similar levels found in the diets (Table 2). The concentration of the oxidation products 4-HHE and 4-HNE in eWAT and rWAT was similar among groups, in the range 0.5 to 2 nmol per g.

### PL-ω3 and TG-ω3 in high-fat diet affect adipocyte size distribution in WAT differently

**Figure 4:**
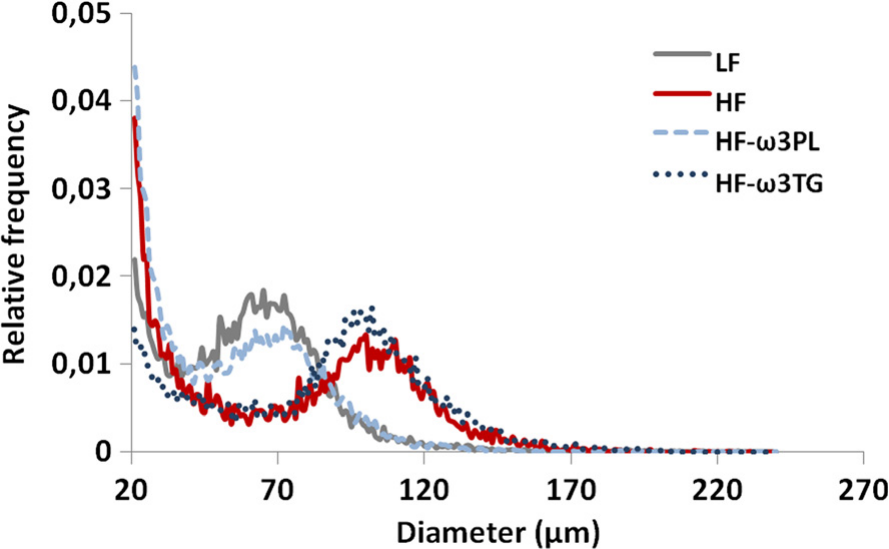
**Frequency evolution of adipocyte size of mice in epididymal fat pad.** Individual measurements were performed on 12 000–14 000 adipocytes using osmium tetroxide - coulter counter procedure as described in Methods. Note that distribution of adipocyte size was shifted leftward (ie. Towards smaller size) in HF-ω3PL mice compared to HF and HF-ω3TG mice. Value are shown for one representative curve for each group.

To know whether the high-fat diets could affect the size and the number of adipocytes in mice, we performed a cellularity analysis of WAT. As shown Figure 4, the size distribution of the epididymal adipocytes in the HF-ω3PL group showed a marked shift toward smaller sizes compared with HF and HF-ω3TG groups. The same effect was observed in retroperitoneal fat pads. The cellular characteristics of eWAT in mice fed HF and HF supplemented with LC *n*-3 PUFA in the form of PL or TG are shown in Table 5. A reduction of fat accretion in eWAT was observed in HF-ω3PL group *vs* HF group but no difference was observed between HF-ω3TG and the two other groups. Adipocyte sizes in HF-ω3PL and LF groups were actually similar. This reduction resulted from a decrease in adipose cell volume rather than from a decrease in the total number of adipocytes per fat pad, as calculated from the measured size distribution. Indeed, mean adipocyte diameter was reduced by 14% in HF-ω3PL group *vs* HF group (*P* < 0.05) resulting in a 30% decrease in calculated adipose cell volume (*P* = 0.03).

**Table 5:** Comparison of adipose cell size variables in eWAT of LF, HF, HF-ω3PL and HF-ω3TG mice

	**LF**	**HF**	**HF-ω3PL**	**HF-ω3TG**
**eWAT**				
eWAT (mg)	502 ± 29	588 ± 69	423 ± 21^*^	536 ± 59
Mode (μm)	77.3 ± 3.9^*^	89.3 ± 6.0	75.6 ± 1.8^*£^	84.9 ± 5.5
Cell diameter (μm)	67.1 ± 3.1^#^	74.6 ± 3.4	64.3 ± 3.0^*^	70.5 ± 4.1
Cell weight (ng)	455.5 ± 46.5^#^	619.9 ± 49.2	434.8 ± 42.9^*^	496.5 ± 99.8
Nb cells (x10^6^)	1.19 ± 0.01	1.12 ± 0.14	1.03 ± 0.07	0.99 ± 0.07

(*P < 0.05 *vs* HF); (^#^P = 0.07 *vs* HF); (^£^P < 0.05 *vs* HF-ω3TG).

Data are mean ± SEM for n = 4-5 per group. Abbreviations: eWAT, epididymal white adipose tissue.

### Effect of PL-ω3 and TG-ω3 in high-fat diet on oxidative stress in the small intestine

**Figure 5:**
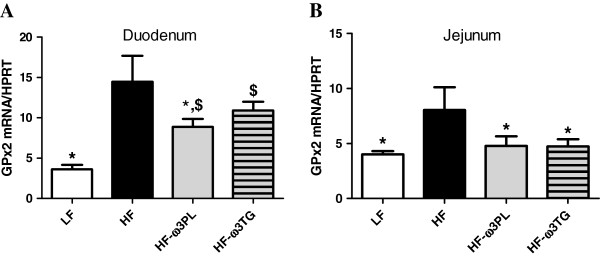
**Expression of gastro-intestinal glutathione peroxidase 2 (GPx2) RNA in the small intestine. **(**A**) duodenum and (**B**) jejunum of mice fed LF, HF, HF-ω3PL and HF-ω3TG diets during 8 weeks. This analysis was quantified by qPCR. Bars represents means ± SEM of n = 5-6 mice. (*P < 0.05 *vs* HF); ANOVA followed by Fisher test.

The upper small intestine represents the primary defense line of the organism that can affect metabolism and inflammation. We thus examined the gene expression of gastrointestinal glutathione peroxidase 2 (GPx2), mainly expressed in the small intestine and implicated in the detoxification of lipid oxidation products including 4-HHE and 4-HNE. The expression of GPx2 in the duodenum was higher in HF mice than in LF and HF-ω3PL groups (Figure 5A). In the jejunum, the gene expression of GPx2 was significantly increased in HF group compared with the three other groups (Figure 5B).

## Discussion

In the present study, we investigated (i) the effects of long-term consumption of HF diet supplemented with LC *n*-3 PUFA in the form of PL or TG on adiposity, oxidative stress and inflammation and (ii) whether the PL or TG carrier can affect these parameters. For this purpose, we designed a HF diet containing 20% of fat, rich in saturated fatty acids and with a relatively high *n*-6/*n*-3 ratio to mimic the lipid-enriched foods as consumed in typical Western diets. Importantly, all diets contained the same amount of PL, in the form of PL-DHA in HF-ω3PL diet and in the form of lecithin PL-LA in all other groups. This formulation ensured that the possible observed effects of PL-ω3 can be attributed to the location of *n*-3 PUFA in the diet (on PL rather than TG) and not to the presence of polar lipids that would have biased the interpretation.

We show that altogether, long-term intake of HF diet supplemented with LC *n*-3 PUFA protects against inflammation and oxidative stress induced by HF diets. This is in agreement with numerous studies documenting the beneficial health effects of LC *n*-3 PUFA [19,37]. In this study, the supplementation of HF diet with LC *n*-3 PUFA in the PL carrier was slightly lower than that in the TG carrier. Therefore, our results indicate that the effects of LC *n*-3 PUFA from lecithin rich in PL-DHA is more efficient than LC *n*-3 PUFA from tuna oil (mainly TG-DHA). This supports the results of a previous study reporting that in healthy humans the metabolic effects of krill oil (mainly PL) are similar to those of fish oil but at lower dose of LC *n*-3 PUFA [27]. Importantly in previous studies, the PL carrier was provided by the bulk marine sources, i.e. krill oil [25-28]. Usually, the percentage of PL in these products is 40%, which means that a part of *n*-3 PUFA brought by the krill oil was in fact bound to TG carrier in these studies.

In the present study, a dietary intake of LC *n*-3 PUFA during 8 weeks resulted in an increased incorporation of DHA into plasma, liver and WAT lipids, the FA composition of the organs reflecting the fatty acid profiles of the diets. Regardless of the LC *n*-3 PUFA carrier, the *n*-6/*n*-3 ratio in plasma, liver and WAT of mice fed HF- ω3PL and HF- ω3TG was lower than in the HF diet. The ratio was almost similar in both HF-ω3PL and HF-ω3TG groups, while the HF-ω3TG group showed a significantly higher proportion of DHA than the HF-ω3PL group. A decreased *n*-6/*n*-3 ratio in tissues has been reported to reduce atherosclerosis due to the inhibition of systemic and vascular inflammation in apolipoprotein E-deficient mice. The authors attributed these protective effects to the anti-inflammatory properties of *n*-3 PUFA [38]. In addition, the proportion of EPA in WAT was higher in mice fed the HF diets supplemented with LC *n*-3 PUFA compared with HF, suggesting that the turnover of DHA in eWAT is high. The increase in EPA concentration paralleled the increased level of DHA in HF-ω3TG more than in HF-ω3PL.

LC *n*-3 PUFA are known to reduce metabolic inflammation in human and rodents [39-41]. Our data show that the HF diet induced higher IL-6, MCP-1 levels in plasma and in eWAT than LF diet. Interestingly, no activation of these pro-inflammatory markers was observed in HF-ω3PL and in HF-ω3TG groups, even if the EPA + DHA dose in the HF- ω3PL is lower of that in the HF-ω3TG group. This may indicate that LC *n*-3 PUFA derived from PL provide a better bioavailability and/or bioactivity than those esterified into TG. Our results suggest that LC *n*-3 PUFA, regardless of their molecular form, could inhibit the low-grade inflammation by directly inhibiting macrophage immigration through the inhibition of MCP-1.

Our data are consistent with previous studies reporting that the inflammatory response in WAT induced by HF diet in obese diabetic animals was prevented by the supplementation HF with *n*-3 PUFA either in the form of PL or TG [6,21]. Batetta et al. concluded that such anti-inflammatory effects can be due to observed lower levels of arachidonic acid in membrane phospholipids [24].

Recent studies revealed the complementary role of metabolic endoxotemia in the low-grade inflammation. Laugerette et al. observed that endotoxin transporter LBP was positively correlated with plasma IL-6 in mice fed a palm oil-based high-fat diet, which was reversed using rapeseed oil [42]. Other works evidenced a link between low-grade inflammation or related metabolic disorders and plasma LBP and sCD14 [43,44]. In our study, the HF group presented the highest plasma concentration of LBP, consistently with inflammatory markers. A significant decrease in the level of plasma sCD14 was observed in HF-ω3PL and HF-ω3TG groups. Altogether, our results indicate that the supplementation of HF diet with LC *n*-3 PUFA can lower plasma concentrations of endotoxin transporters. Further mechanisms should be investigated to elucidate the implication of *n*-3 PUFA sources in the regulation of endotoxemia-induced metabolic inflammation.

Circulating leptin levels are directly associated with the mass of WAT and inflammation [45]. Our results showed that the plasma levels of leptin, mainly produced by adipocytes, were decreased in the two supplemented groups with LC *n*-3 PUFA in the form of PL or TG *vs* HF mice. Plasma leptin was also lower in HF-ω3TG group independently of WAT mass, suggesting a relationship between the lower metabolic inflammation and the leptin. Our results are in agreement with a previous study showing that long-term intake of dietary *n*-3 PUFA by rats resulted in a significant decrease in plasma leptin levels [46].

Adiponectin plays an important role as insulin-sensitizing adipokine which production is decreased in obesity and in conditions associated with insulin resistance [5,47]. In our study, we did not observed a significant difference in circulating adiponectin level among groups. In addition, the glucose and insulin tolerances were not affected by the different diets. Our results are in agreement with previous study reporting that the level of adiponectin was not different between HF group (35% of fat) and DHA/EPA in the form of PL or TG supplemented to the HF diets for 8 weeks [21]. Conversely, other studies showed a decrease in the level of plasma adiponectin after feeding mice a HF diet; this level was restored by the supplementation HF diet with DHA and EPA [6,29]. Altogether, such modifications in plasma adiponectin concentrations are reported with different lipid types (*n*-6 PUFA-rich corn oil instead of lard) and/or concentrations in the diet (35% w/w instead of 20%) and using larger doses of fish oil (15-40% in total dietary lipids) compared with the present study (5%).

Regarding adiposity, HF diet did not induce significantly higher body weight gain than the low fat control, although a trend towards heavier adipose tissue was observed. This can be due to our choice of preparing LF and HF groups using similar ingredients. Both LF and HF were semi-synthetic and based mainly on corn starch and casein, while many studies in the literature reach weight gain by comparing semi-synthetic high-fat diet with regular chow based on more various ingredients [48]. Among the HF diets, HF-ω3PL diet led to a lower weight gain and a reduced adipose tissue compared with HF group without affecting the mass of muscles. However these effects were not observed in mice fed HF-ω3TG. These results suggest that the PL carrier can decrease body fat deposition more than the TG carrier. This is consistent with a previous study reporting that obese mice fed LC *n*-3 PUFA carried by PL developed less body weight gain than those fed LC *n*-3 PUFA carried by TG [21]. We further investigated the effect of HF diet containing LC *n*-3 PUFA in the form of PL or TG on the cell size distribution of adipose tissue by using the Coulter counter method (Multisizer IV, Beckman Coulter) which allows a more precise cell-size distribution because the number of cell counted is much higher than the one of microscopic methods. The most noticeable difference between groups was in the adipocyte size distribution. Mice fed HF diet showed an increased adipocyte size compared with those fed the LF diet. Differential effects were observed regarding LC *n*-3 PUFA supplementation; HF-ω3PL and LF diets induced similar adipocyte size whereas HF-ω3TG and HF diets led to larger adipocytes. Our results following a 20% w/w high-fat diet for 8 weeks are consistent with those of Rossmeisl et al. [21]. These authors observed that the PL carrier decreased adipocyte area compared with the control HF diet in obese mice, which was not observed using the TG carrier. Of note, endothelial lipase presents specificity towards DHA-containing PL, thus generating Lyso-PL containing DHA [49]. Moreover, our PL source also contained Lyso-PC-DHA. We may hypothesize that such DHA-containing lipid species might exert specific biological activity, because superior biological effects of LysoPC-DHA have otherwise been demonstrated regarding DHA accretion in the brain and anti-inflammatory activity [50,51]. However, further research is required to better understand the mechanism of action of PL carrier.

Skurk et al. investigated the secretory capacity of adipocyte fractions from the same individual. They demonstrated that the large adipocytes were implicated in the induction of pro-inflammatory genes such as those coding for IL-6 and MCP-1 [52]. Consistently, HF diet-induced-inflammation can be associated with large adipocytes. Regarding the impact of supplementing with *n*-3 PUFA, noticeably, the HF-ω3PL diet had a greater anti-adiposity effect than the HF-ω3TG diet associated with smaller adipocytes. Here, the anti-adiposity effect of HF-ω3PL diet could be partly associated with the lower metabolic inflammation in the mice. In contrast, the anti-inflammatory effect of HF-ω3TG was not associated with a decrease in adiposity.

We further investigated the effect of the composition of the dietary high-fat, and more specifically of the presence of LC *n*-3 PUFA on lipid peroxidation and oxidative stress. LC PUFA are molecules susceptible to oxidation because they contain many double bonds. PUFA oxidation leads to the formation of secondary end-products such as 4-HNE derived from *n*-6 PUFA and 4-HHE derived from *n*-3 PUFA [53]. Our previous studies in mice showed that these markers can induce oxidative stress and inflammation, even when consumed in moderately oxidized dietary fat [30]. They also provoke oxidative stress *in vitro* in Caco-2 cells [30]. In the present study, similar levels of plasma 4-HHE were measured in the three HF mice groups; however the plasma level of 4-HNE was increased in HF mice compared with the other groups. Previously, Esterbauer et al. found that the basal concentrations of 4-HNE in the human serum were in the range 0–700 nM. In our study, plasma 4-HNE in mice remained within this range observed in humans [54]. In liver and in WAT, the levels of 4-hydroxy-2-alkenals were lower than 2 nmol/g, which is in the same order of magnitude than in another study reporting basal concentrations of 4-HNE in the liver of mice [55]. We thus suggest that the dietary high-fat used in this study may slightly enhance the oxidative stress through the induction of 4-HNE in plasma, albeit without increase of 4-HNE in tissues. LC *n*-3 PUFA supplementation prevented this phenomenon.

We propose that the increase of oxidative stress after HF diet consumption is linked to an alteration of anti-oxidant defenses. In the gastro-intestinal tract, a defense system including GPx2, is able to detoxify lipid peroxidation products and protect against inflammation [30,56,57]. Our results show a significant increase of GPx2 mRNA in the duodenum of mice fed HF *vs* HF-ω3PL diets. Interestingly, a significant difference of GPx2 mRNA in the jejunum was also observed between HF *vs* HF-ω3PL mice. Meanwhile, we observed a significant difference between HF *vs* HF-ω3TG groups in the jejunum. This suggests that (i) the HF diet-induced-inflammation could alter the anti-oxidant defense system in the intestine and (ii) the supplementation of HF diet with LC *n*-3 PUFA in the form of PL is more bioactive in the upper intestine than those in the form of TG.

Regarding the concentration of α-tocopherol, a lipid-soluble antioxidant vitamin, we show that it was significantly increased in the WAT of HF-ω3PL and HF-ω3TG groups as compared to HF mice. Interestingly, tocopherol level in WAT was significantly greater in HF-ω3PL than in HF-ω3TG mice. Of note, in this study we took care to adjust dietary tocopherol to obtain similar concentrations in our diets. Thus, our results can be attributed to the direct effects of PL-bound *vs* TG-bound LC *n*-3 PUFA. Consistently, Choi et al. recently reported that high-fat diet enhanced the oxidative stress through the decreased level of tocopherols in the livers of rats [58]. Thus, our findings suggest that dietary supplementation with LC *n*-3 PUFA is beneficial for decreasing lipid peroxidation in high fat-fed mice, and that the PL carrier may induce a superior bioavailability to tocopherols or lower need for their use to counteract oxidative stress. The enhanced level of α- tocopherol following LC *n*-3 PUFA in the form of PL can also be an effective defense against oxidative stress and inflammation. Further studies should analyze the amount and distribution of vitamin E among different tissues after the diets. The effect of dietary *n*-3 PUFA in the form of PL and TG on the activity of anti-oxidant systems should also be clarified by further studies in humans. Altogether, HF diet induced concomitant increase in plasma 4-HNE, GPx-2 activation in the small intestine and lower tocopherol level in WAT; all of these markers being reversed by the supplementation with LC *n*-3 PUFA.

## Conclusion

In conclusion, our study demonstrates in mice that long-term ingestion of *n*-3 PUFA prevents HF diet-induced inflammation and oxidative stress. Compared with triacylglycerols, LC *n*-3 PUFA supplemented in the form of PL exhibit superior beneficial metabolic effects by decreasing adiposity, reducing adipocyte size and stimulating the anti-oxidant system. Our findings should be strengthened by dietary intervention studies in human aimed at testing the impact of different LC *n*-3 PUFA molecular forms on the structure and metabolism of adipose tissue. Such findings could support the development of functional foods containing LC *n*-3 PUFA in the form of PL participating to the prevention of the development of chronic metabolic diseases in humans like obesity.

## Abbreviations

PUFA: Polyunsaturated fatty acids; LF: Low fat; HF: High-fat; DHA: Docosahexaenoic acid; EPA: Eicosapentaenoic acid; TG: Triacylglycerols; PL: Phospholipids; WAT: White adipose tissue; 4-HHE: 4-hydroxy-2-hexenal; 4-HNE: 4-hydroxy-2-nonenal; GPx2: Gastrointestinal glutathione peroxidase 2; IL: Interleukin; MCP-1: Monocyte chemotactic protein-1; LBP: Lipopolysaccharide binding protein; sCD14: Soluble cluster of differentiation.

## Competing interests

The authors have no competing interests to disclose.

## Authors’ contribution

MA designed research, performed experiments, interpreted data and wrote the manuscript. COS, AM, LH, AG, MV and CD performed experiments, interpreted data and helped edit the manuscript. BB and LR performed experiments. ML and MG contributed to manuscript preparation. CG supervised the project and helped edit the manuscript. MCM supervised the work, designed research, interpreted data and wrote the manuscript. All authors read and approved the final manuscript.

## Supplementary Material

Additional file 1 Fatty acid composition in tuna oil and in purified PL-DHA. Click here for file

Additional file 2 Fatty acid profile in liver and eWAT of mice fed different diets. Click here for file

Additional file 3 Fatty acid profile in rWAT in mice fed different diets. Click here for file

Additional file 4 MCP1 mRNA level in retroperitoneal white adipose tissue (rWAT). Click here for file
