# Supplementary material for: n-3 PUFA added to high-fat diets affect differently adiposity and inflammation when carried by phospholipids or triacylglycerols in mice
Source: Nutr Metab (Lond). 2013 Feb 15;10:23. doi: 10.1186/1743-7075-10-23 (PMC3585798; doi:10.1186/1743-7075-10-23)
Supplement: Additional file 1 — Fatty acid composition in tuna oil and in purified PL-DHA. [file 1743-7075-10-23-S1.docx]

|  | Tuna oil | | Purified PL-DHA | |
| --- | --- | --- | --- | --- |
|  | % | mg/g lipid | % | mg/g lipid |
| 14:0 | 3.9 ± 0.4 | 25.3 ± 3.8 | 0.1 ± 0.0 | 0.35 ± 0.1 |
| 15:0 | 1.2 ± 0.1 | 7.6 ± 0.3 |  |  |
| 16:0 | 22.5 ± 0.9 | 145.7 ± 7.8 | 3.7 ± 0.2 | 17.4 ± 1.8 |
| 18:0 | 6.5 ± 0.1 | 41.8 ± 2.7 | 2.0 ± 0.1 | 9.4 ± 1.0 |
| 20:0 | 0.4 ± 0.0 | 2.6 ± 0.3 | 0.1 ± 0.0 | 0.4 ± 0.1 |
| SFA | 34.9 ± 1.2 | 222.9 ± 13.4 | 6.3 ± 0.3 | 29.1 ± 2.2 |
| 16:1 *n*-9 | 0.8 ± 0.1 | 5.4 ± 0.3 | 0.05 ± 0.02 | 0.2 ± 0.1 |
| 16:1 *n*-7 | 4.8 ± 0.2 | 30.8 ± 1.9 | 0.3 ± 0.0 | 1.5 ± 0.1 |
| 18:1 *n*-9 | 17.9 ± 0.3 | 115.6 ± 7.1 | 20.5 ± 0.5 | 95.6 ± 10.8 |
| 20:1 | 1.5 ± 0.0 | 9.9 ± 0.7 | 0.4 ± 0.2 | 1.8 ± 0.9 |
| 24:1 | 0.4 ± 0.1 | 2.5 ± 0.6 | 0.1 ± 0.0 | 0.3 ± 0.0 |
| MUFA | 25.2 ± 0.7 | 161.7 ± 9.5 | 22.1 ± 0.4 | 103.1 ± 11.7 |
| 18:2 *n*-6 | 2.0 ± 0.2 | 13.0 ± 1.5 | 11.2 ± 0.2 | 52.0 ± 6.0 |
| 20:2 *n*-6 | 0.3 ± 0.1 | 2.0 ± 0.7 | 0.3 ± 0.0 | 1.4 ± 0.1 |
| 20:3 *n*-6 | 0.2 ± 0.0 | 1.0 ± 0.1 | 0.4 ± 0.0 | 1.7 ± 0.2 |
| 20:4 *n*-6 | 2.6 ± 0.1 | 17.0 ± 1.4 | 3.9 ± 0.0 | 18.2 ± 2.1 |
| *n*-6 PUFA | 5.1 ± 0.2 | 33.0 ± 3.0 | 18.2 ± 0.2 | 84.8 ± 9.5 |
| 18:3 *n*-3 | 1.0 ± 0.7 | 6.6 ± 4.5 | 1.2 ± 0.0 | 5.7 ± 0.6 |
| 20:4 *n*-3 | 0.5 ± 0.2 | 3.4 ± 1.3 | 0.6 ± 0.0 | 2.6 ± 0.4 |
| 20:5 *n*-3 | 6.9 ± 0.2 | 44.6 ± 3.9 | 8.8 ± 0.1 | 41.0 ± 5.0 |
| 22:5 *n*-3 | 1.2 ± 0.1 | 7.7 ± 0.8 | 5.3 ± 0.1 | 24.7 ± 2.5 |
| 22:6 *n*-3 | 26.1 ± 1.1 | 169.0 ± 17.8 | 38.0 ± 0.8 | 177.3 ± 23.0 |
| *n*-3 PUFA | 35.2 ± 1.6 | 231.3 ± 23.3 | 54.1 ± 0.9 | 252.6 ± 31.8 |
| Total PUFA | 40.3 ± 1.7 | 264.3 ± 26.0 | 72.3 ± 1.0 | 337.4 ± 41.3 |
| *n*-6/*n*-3 ratio | 0.14 ± 0.01 |  | 0.34 ± 0.00 |  |
| Quantity of fatty acids |  | 646.4 ± 46.3 |  | 461.9 ± 48.8 |
| Total tocopherols µg g^-1^ lipids | 851 ± 25 |  | traces |  |

**Additional file 1. Fatty acid composition both in tuna oil and in purified PL-DHA**

**Abbreviations: MUFA, monounsaturated fatty acids; SFA, saturated fatty acids;** PUFA: polyunsaturated fatty acids**.**
