# Supplementary material for: n-3 PUFA added to high-fat diets affect differently adiposity and inflammation when carried by phospholipids or triacylglycerols in mice
Source: Nutr Metab (Lond). 2013 Feb 15;10:23. doi: 10.1186/1743-7075-10-23 (PMC3585798; doi:10.1186/1743-7075-10-23)
Supplement: Additional file 2 — Fatty acid profile in liver and eWAT of mice fed different diets. [file 1743-7075-10-23-S2.docx]

**Additional file 2. Fatty acid profile in liver and eWAT of mice fed different diets.**

|  | Liver | | | | eWAT | | | |
| --- | --- | --- | --- | --- | --- | --- | --- | --- |
| Major FA (mol/100 mol FA): | LF | HF | HF-ω3PL | HF-ω3TG | LF | HF | HF-ω3PL | HF-ω3TG |
| SFA | 33.3 ±0.5^$^ | 31.6 ±0.6 | 36.0 ±0.6^*$^ | 34.6 ±0.4^$^ | 22.7 ±1.9 | 23.5 ±1.8 | 23.5 ±2.6 | 25.8 ±2.5 |
| 16:1 *n*-7 | 3.4 ±0.3 | 1.5 ±0.2^$^ | 1.2 ±0.1^$^ | 1.8 ±0.1^$^ | 7.7 ±0.8 | 5.3 ±0.5^$^ | 5.1 ±1.2^$^ | 5.3 ±0.9^$^ |
| 18:1 *n*-7 | 3.8 ±0.3 | 2.7 ±0.2 | 1.7 ±0.1 | 03.0 ±0.1 | 3.2 ±0.3 | 3.0 ±0.1 | 2.7 ±0.2 | 2.9 ±0.1 |
| 18:1 *n*-9 | 21.9 ±2.7 | 25.1 ±1.9^*^ | 20.4 ±1.2^*^ | 21.6 ±1.3^*^ | 44.8 ±0.5 | 49.8 ±0.7^$^ | 49.3 ±1.1^$^ | 48.2 ±0.4^$^ |
| MUFA | 34.1 ±1.3 | 34.5 ±1.7 | 27.1 ±1.2 | 30.2 ±1.3 | 58.3 ±0.4 | 60.5 ±0.6 | 59.7 ±0.7 | 58.7 ±0.4 |
| 18:2 *n*-6 | 12.1 ±0.4 | 12.6 ±0.2 | 13.6 ±0.4 | 12.1 ±0.2 | 16.9 ±0.7 | 14.7 ±0.3 | 15.0 ±1.4 | 13.7 ±0.7 |
| 20:4 *n*-6 | 11.1 ±0.4 | 11.9 ±0.5 | 10.1 ±0.4 | 9.0 ±0.4 | 0.3 ±0.0 | 0.2 ±0.0 | 0.3 ±0.0 | 0.2 ±0.0 |
| *n*-6 PUFA | 24.7 ±0.7 | 27.3 ±0.7^$^ | 26.8 ±0.4^$^ | 23.4 ±0.5 | 17.2 ±07 | 14.9 ±0.3 | 15.3 ±1.4 | 14.3 ±0.7 |
| 18: 3 *n*-3 | 0.4 ±0.0^$^ | 0.2 ±0.0 | 0.3 ±0.0 | 0.3 ±0.0 | 1.4 ±0.1 | 0.5 ±0.0^$^ | 0.7 ±0.1^$^ | 0.6 ±0.0^$^ |
| 20:5 *n*-3 | 0.7 ±0.0 | 0.4 ±0.0^£^ | 0.4 ±0.0^$£^ | 0.8 ±0.0^*^ | Tr | Tr | 0.1 ±0.0^*$£^ | 0.2 ±0.0^$*^ |
| 22:6 *n*-3 | 6.0 ±0.3 | 5.3 ±0.4 | 8.8 ±0.3*^*$£^* | 10.2 ±0.4*^*$^* | Tr | Tr | 0.2 ±0.0^*$£^ | 0.4 ±0.0^$*^ |
| *n*-3 PUFA | 7.9 ±0.4 | 6.6 ±0.4 | 9.9 ±0.3*^$*^* | 11.7 ±0.4*^$*^* | 1.5 ±0.1 | 0.7 ±0.1^$^ | 1.1 ±0.1 | 1.2 ±0.1 |
| *n*-6/*n*-3  ratio | 3.2 ±0.1^*^ | 4.2 ±0.2 | 2.7 ±0.1^*^ | 2.0 ±0.4^*^ | 11.8 ±0.3^*^ | 23.3 ±1.6 | 14.2 ±0.9^*^ | 12.4 ±0.8^*^ |

**(**P*<0.05 *vs* HF);** (^$^*P*<0.05 *vs* LF); **(^£^*P*<0.05 *vs* HF-ω3TG). Data are mean ±SEM for n=5-7 per group for eWAT and n=8-9**

**per group for liver. Abbreviations: Tr, traces; eWAT:** [epididymal](http://en.wikipedia.org/w/index.php?title=Epididymal&action=edit&redlink=1) [white adipose tissue](http://en.wikipedia.org/wiki/White_adipose_tissue); **FA, fatty acids;**

**MUFA, monounsaturated fatty acids; SFA, saturated fatty acids**; PUFA: polyunsaturated fatty acids**.**
