# Supplementary material for: n-3 PUFA added to high-fat diets affect differently adiposity and inflammation when carried by phospholipids or triacylglycerols in mice
Source: Nutr Metab (Lond). 2013 Feb 15;10:23. doi: 10.1186/1743-7075-10-23 (PMC3585798; doi:10.1186/1743-7075-10-23)
Supplement: Additional file 3 — Fatty acid profile in rWAT in mice fed different diets. [file 1743-7075-10-23-S3.docx]

**Additional file 3 Fatty acid profile in rWAT in mice fed different diets.**

| Major FA in rWAT  Total FA (mol/100 mol FA): | LF | HF | HF-ω3PL | HF-ω3TG |
| --- | --- | --- | --- | --- |
| SFA | 22.6 ±0.9 | 25.3 ±1.5 | 25.1 ±1.2 | 26.1 ±1.1 |
| 16:1 *n*-7 | 8.5 ±0.8 | 5.0 ±0.6^$^ | 4.2 ±0.3^$^ | 5.7 ±0.2^$^ |
| 18:1 *n*-7 | 3.3 ±0.4 | 2.9 ±0.3 | 3.0 ±0.1 | 3.0 ±0.1 |
| 18:1 *n*-9 | 47.2 ±1.3 | 51.5 ±1.5^$^ | 52.8 ±1.2^$^ | 50.3 ±0.8^$^ |
| MUFA | 61.9 ±0.1 | 62.0 ±1.4 | 63.2 ±1.3 | 61.6 ±0.8 |
| 18:2 *n*-6 | 13.6 ±0.9 | 11.5 ±0.4 | 10.1 ±0.2^$^ | 10.8 ±0.4^$^ |
| 20:4 *n*-6 | 0.2 ±0.0 | 0.2 ±0.0 | 0.2 ±0.1 | 0.2 ±0.0 |
| *n*-6 PUFA | 14.2 ±0.8 | 12.2 ±0.4 | 10.8 ±0.3^$^ | 11.4 ±0.5^$^ |
| 18: 3 *n*-3 | 1.2 ±0.1 | 0.5 ±0.1^$^ | 0.5 ±0.1^$^ | 0.5 ±0.0^$^ |
| 20:5 *n*-3 | Tr^£^ | Tr^£^ | Tr^£^ | 0.1 ±0.0 |
| 22:6 *n*-3 | Tr | Tr | 0.1 ±0.1*^*$£^* | 0.3 ±0.1*^*$^* |
| *n*-3 PUFA | 1.3 ±0.0 | 0.6 ±0.0^$^ | 0.8 ±0.1*^$*^* | 1.0 ±0.0*^$*^* |
| *n*-6/*n*-3 ratio | 11.1 ±0.2^*^ | 21.7 ±1.6 | 13.9 ±1.4^*^ | 11.8 ±0.6^*^ |

(*P<0.05 *vs* HF); (^$^P<0.05 *vs* LF); (^£^P<0.05 *vs* HF-ω3TG). Data are mean ±SEM for *n*=4-5 per group. Abbreviations: Tr, traces; rWAT, [retroperitoneal](http://en.wikipedia.org/w/index.php?title=Epididymal&action=edit&redlink=1) [white adipose tissue](http://en.wikipedia.org/wiki/White_adipose_tissue) ; FA, fatty acids; MUFA, monounsaturated fatty acids; SFA, saturated fatty acids; PUFA: polyunsaturated fatty acids.
