# Supplementary material for: n-3 PUFA added to high-fat diets affect differently adiposity and inflammation when carried by phospholipids or triacylglycerols in mice
Source: Nutr Metab (Lond). 2013 Feb 15;10:23. doi: 10.1186/1743-7075-10-23 (PMC3585798; doi:10.1186/1743-7075-10-23)
Supplement: Additional file 4 — MCP1 mRNA level in retroperitoneal white adipose tissue (rWAT). [file 1743-7075-10-23-S4.docx]

**Additional file 4. MCP1 mRNA level in** [**retroperitoneal**](http://en.wikipedia.org/w/index.php?title=Epididymal&action=edit&redlink=1) [**white adipose tissue**](http://en.wikipedia.org/wiki/White_adipose_tissue) **(rWAT)**

Bars represents means ± SEM of n=5-6 mice. (**P*<0.05). ANOVA followed by Fisher test.
